# A city park as a potential epidemic site of scrub typhus: a case–control study of an outbreak in Guangzhou, China

**DOI:** 10.1186/s13071-014-0513-7

**Published:** 2014-11-18

**Authors:** Yuehong Wei, Lei Luo, Qinlong Jing, Xiaoning Li, Yong Huang, Xincai Xiao, Lan Liu, Xinwei Wu, Zhicong Yang

**Affiliations:** Guangzhou Center for Disease Control and Prevention, Guangzhou, Guangdong Province China; School of Public Health, Guangdong Pharmaceutical University, Guangzhou, Guangdong Province China

**Keywords:** City, Park, Scrub typhus, Epidemiology

## Abstract

**Background:**

Scrub typhus is an important public health problem in China, especially in Guangzhou city. Typical outbreaks of scrub typhus have been previously reported in rural areas, affecting mainly farmers. We describe an atypical outbreak of the disease with case fatalities, from a park in Haizhu District, Guangzhou, that could turn out to be a potential scrub typhus epidemic site.

**Methods:**

From May 2012 to June 2012, a case–control study was conducted to identify source and risk factors of this outbreak. Reported cases of scrub typhus in Xiaogang Park were confirmed by Weil–Felix test or a nested polymerase chain reaction (NPCR). Controls were matched with their neighbors by gender and age. Multivariate conditional logistic regression was used to identify risk factors and protective factors.

**Results:**

A total of 29 cases were confirmed by Weil–Felix test, including 4 deaths by both Weil–Felix test and NPCR. All patients presented with fever (100%), while 28 (96.6%) cases had eschars, 10 (34.5%) headache, 10 (34.5%) chills, 6 (20.7%) lymphadenopathy, 5 (17.2%) rash, 2 (6.9%) vomiting and 1 (3.5%) presented with conjunctival congestion. The proportion of cases with activity history in Xiaogang Park was much higher than the control group (72.4% vs 24.1%, P < 0.001), and morning exercise in park or field was also as a risk factor for scrub typhus (adjusted OR = 3.0, 95% CI: 1.1 - 8.2). Four factors were significantly associated with the risk of developing scrub typhus: sitting on the lawn (adjusted OR = 8.0, 95% CI: 1.4 - 44.5), close contact with rats (adjusted OR = 3.3, 95% CI: 1.2 -9.6), sitting near the rat holes (OR = 6.8, 95% CI: 1.2 - 38.1) and wearing long-sleeved clothing when outside (adjusted OR = 0.3, 95% CI: 0.1 - 0.7).

**Conclusions:**

We confirmed an atypical outbreak of scrub typhus in a park in Guangzhou city, which has the potential to develop into an important epidemic site. This public health risk should not be neglected and requires more attention from authorities.

## Background

Scrub typhus (Tsutsugamushi disease) is a febrile illness caused by the *rickettsial bacterium, Orientia tsutsugamushi*. It is transmitted by the bites of infected chiggers of the *Trombiculidae* family, especially those of the genus *Leptotrombidium* [1]. Clinical presentation in patients varies from an asymptomatic to a life-threatening disease, including acute respiratory distress syndrome and acute liver failure [2]. To date, no effective and reliable human vaccine against scrub typhus is available [3].

The epidemic occurs mainly in Asia and the Pacific [4–7], wherein Japan, Korea and China are renowned as ‘the triangle of scrub typhus’ [8–11]. It is estimated that over half (55%) of the world’s population live in scrub typhus endemic areas, and that about one million cases occur annually [12].

As a natural epidemic focus, the habitat conditions of scrub typhus include weeds along rivers, shrub, grassland and abandoned farmlands. People engaged in agriculture and forestry are at high risk for scrub typhus. In recent decades, with the economic development [2], population movement, urbanization [13], climatic changes and the overutilization of natural resources, the epidemic focus is expanding continuously [14]. The incident population have spread from occupational population to general population such as the elderly, children, pregnant women and tourists [2,15–17].

In 1948, the first Chinese case of scrub typhus case was reported in Guangzhou, and was later spread to other provinces, including Shandong, Anhui and Beijing [1,5,14]. Due to its severity, scrub typhus is a reportable disease in China. A total of 27,391 confirmed cases of scrub typhus were reported in China during 2006–2012, with southeastern and southwestern China reporting higher numbers [8]. From 2006 to 2012 Guangzhou reported a total of 3997 confirmed cases with an obvious increase of reported cases in 2012.

In China, outbreaks of scrub typhus are typically reported in rural areas, and it is primarily farmers are who are infected. To the best of our knowledge, there is no previous report describing citizens acquiring scrub typhus from an urban park. From May 2012, a large number of patients with fever, headache, lymphadenopathy and rash were discovered from our surveillance system, 4 fatal cases were diagnosed as scrub typhus. As scrub typhus seldom causes death in Guangzhou, it attracted our attention. Here, we confirm an atypical outbreak of scrub typhus with fatal cases through use of laboratory diagnosis and epidemiological investigation. This outbreak occurred in a park in Haizhu District, Guangzhou, China, which has potential to become a scrub typhus epidemic focus due to its usage by the public.

## Methods

### Study area

**Figure 1:**
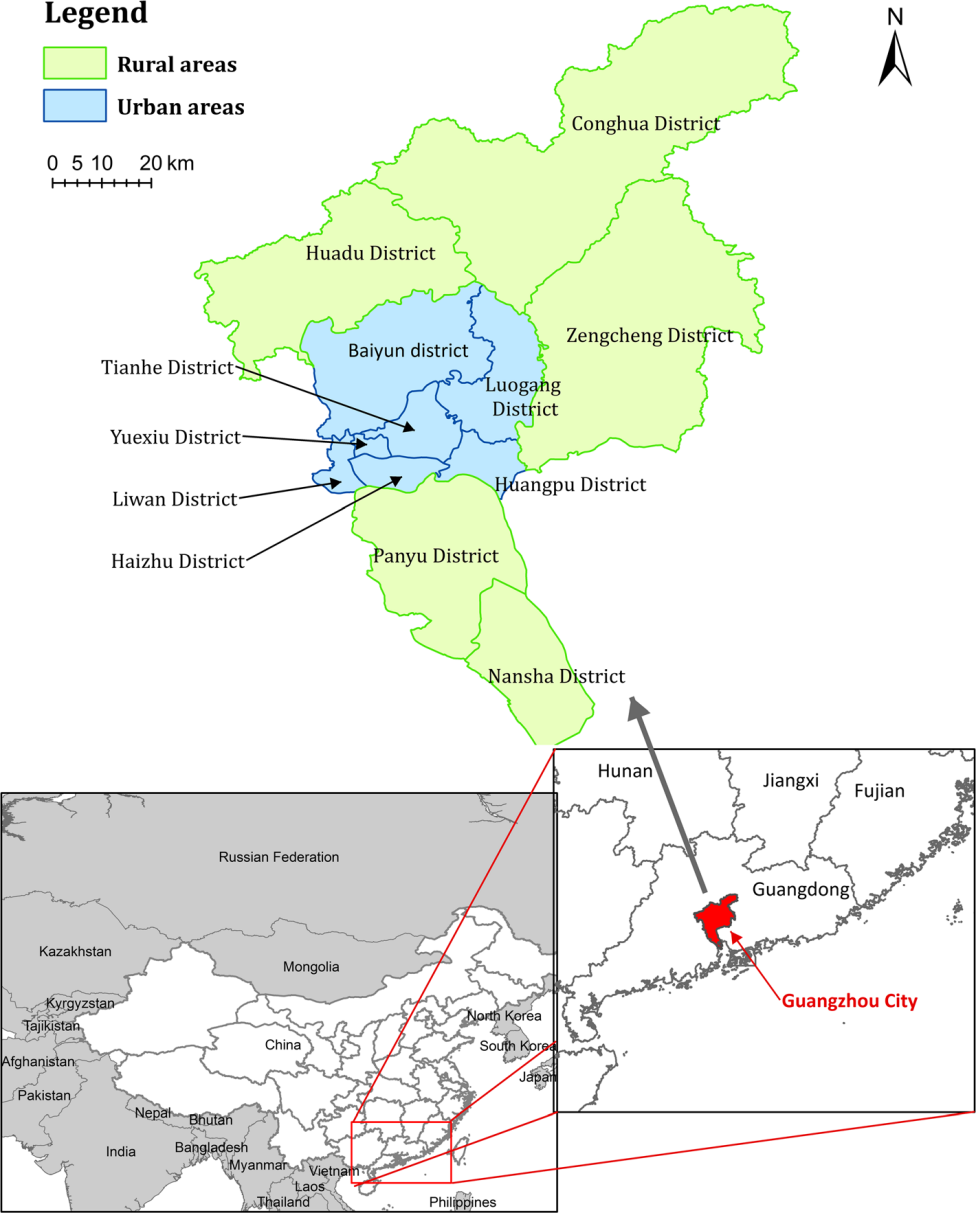
**Geographic location of Guangzhou City, Guangdong Province, China.**

Guangzhou (Figure 1) is the capital of Guangdong province of China, It is located between longitudes 112°57'E and 114°3'E, latitudes 22°26'N and 23°56'N. The city population in 2010 was 12.70 million [18]. It is influenced by a subtropical monsoon climate, yielding hot rainy summers and mild winters. The average January temperature is about 0°C, while the corresponding July temperature hovers around 25°C. The annual rainfall is between 1500 mm to 2000 mm and occurs mainly in summer. There is subtle difference in humidity between summer and winter due to a considerable amount of precipitation in winter. Subtropical evergreen broad-leaved forest is the main vegetation of Guangzhou, and the green coverage rate is 24.57%.

**Figure 2:**
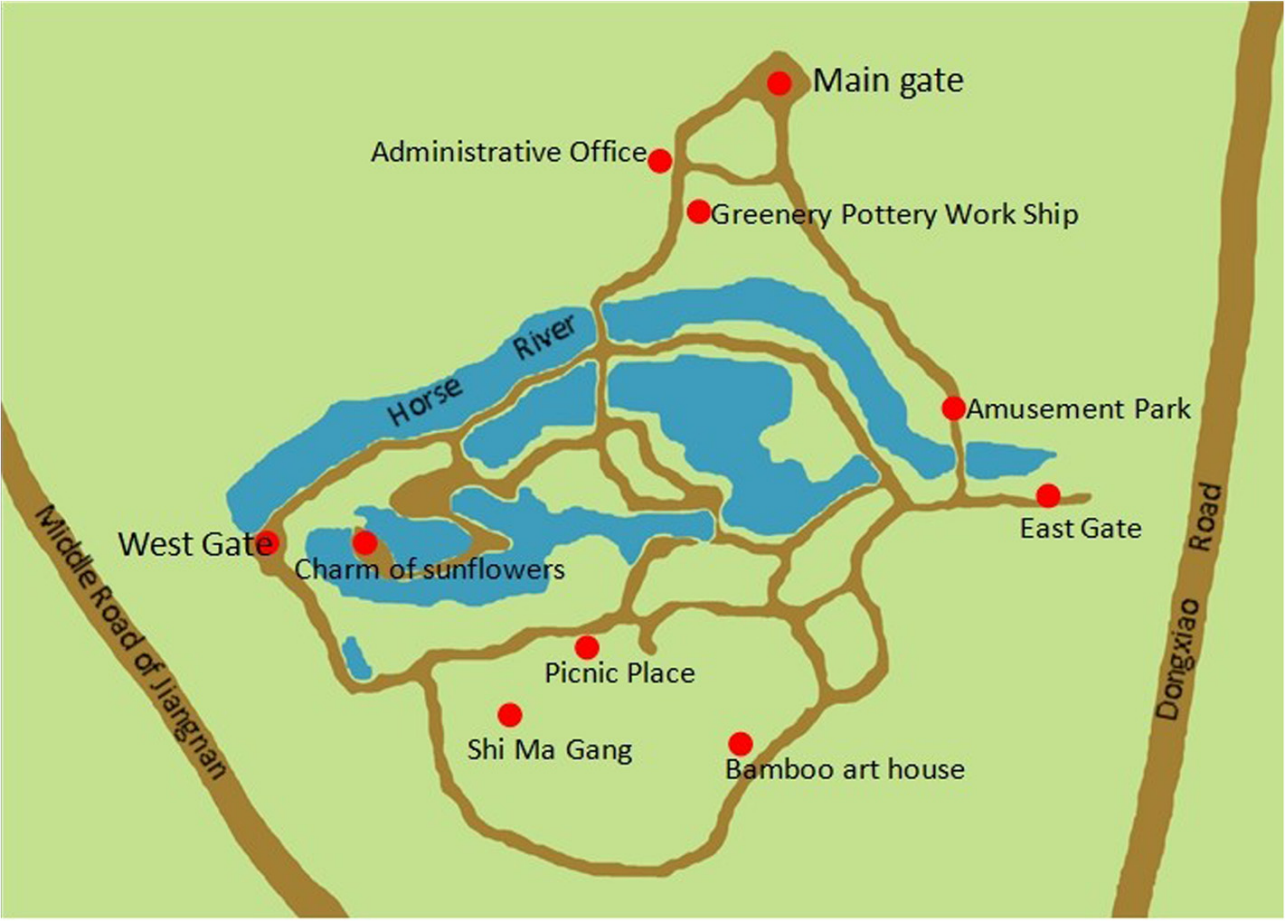
**Geographic location of Xiaogang Park.**

Xiaogang Park (Figure 2) with an area of 167,000 m^2^, is located in the southwest of Haizhu District, Guangzhou and has a wide grass coverage. In the park, the Greenery pottery workshop, Picnic place and Shi Ma Gang are the places with the most people-related activity. Between 19 to 26 May 2012, 4 fatal cases with fever, headache, eschar, lymphadenopathy and rash were discovered in Haizhu District, Guangzhou. Case survey showed that they all had history of activity in Xiaogang Park, so we conducted research in that area by a questionnaire in order to identify the association between the outbreak and the park.

### Study design

A retrospective case–control study was designed for identifying the source of this outbreak. A case was defined as any scrub typhus patient reported between 1 May and 20 June in Haizhu district. For every case, four controls were sought in the neighborhood. These controls were of the same gender, within 2 years’ age difference, and lived in the same building or within 50 meters away from the respective case. Trained investigators visited and interviewed both the cases and controls with the same structured questionnaire. The questionnaire consisted of five parts: social and demographic characteristics (age, gender, occupation and residence), clinical features (onset date, history of disease, complication, fever, rash, eschar, chills, headache, conjunctival congestion and vomiting), history of outdoor activities within one month from onset (travelling, morning exercise and activity history in Xiaogang Park), potential risk factors (sitting on the lawn, fishing, close contact with cats, close contact with rats, sitting next to rat holes, keeping dogs and cats) and potential protective factors of scrub typhus (wearing long-sleeved clothing when outside and using insect repellent).

### Diagnosis and laboratory investigation of cases

The diagnostic criteria for scrub typhus were based on a guidebook published by the Chinese Center for Disease Control and Prevention (China CDC) [19]. Patients with two of the following were defined as clinically diagnosed cases: (i) field exposure history 1–3 weeks prior to onset of symptoms, (ii) sudden high fever accompanied by characteristic eschar or ulcer, (iii) enlarged lymph nodes, skin rash, splenomegaly, or hepatomegaly. The confirmed scrub typhus case was defined as: agglutination titer >1:160 in the Weil–Felix test using the OX-K strain of *Proteus mirabilis*, or a positive 56-kD nested polymerase chain reaction (NPCR) test. For the NPCR test [20], DNA was extracted from blood clots using a blood DNA extraction kit (Invitrogen, China). The *Orientia tsutsugamushi* 56-kDa protein gene was amplified by nested PCR. The outside primer pair comprised TACATTAGCTGCGGGTATGACA and CCAGCATAATTCTTCAACCAAG. The nested primer pair comprised GAGCAGAGCTAGGTGTTATGTA and TAGGCATTATAGTAGGCTGAGG. PCR products were 306 to 339 bp for the outside primer pair and 150 to 168 bp for the nested primer pair. PCR conditions were the same for both primer pairs, with initial denaturation for 5 min at 94°C, followed by 35 cycles of 1 min at 94°C, 1 min at 50°C, and 1 min at 72°C, and a final extension of 5 min at 72°C. PCR products were separated by electrophoresis, stained with ethidium bromide, and recorded by using the BioRad gel imaging system.

### Informed consent and ethical issues

We obtained verbal informed consent from all study subjects. This study and the use of oral consent procedures were reviewed and approved by Guangzhou Center for Disease Control and Prevention Ethics Committee and Guangzhou Health Bureau.

### Statistic analysis

Data was entered using Epidata 3.1(Epidata Association, Odense, Denmark) and SPSS 20.0 (IBM SPSS, Chicago, Illinois) was used for statistic analysis. Quantitative data were described by means and standard deviation, while socioeconomic status of cases and controls was compared by conditional logistic regression. Multivariate conditional logistic regression was used to evaluate the potential risk factors and protective factors. The crude odds ratios (ORs) and adjusted ORs (adjusted for age, and gender) and their 95% confidence intervals (CI) were reported.

## Results

We identified a total of 29 scrub typhus cases. The first case was reported on 1 May 2012 and the last case was reported on 17 June 2012. Fourteen (48.3%) patients were men. The age of patients ranged from 24 to 86 years, with a median age of 58 years. There were four fatal cases that included one male and three females, all over 70 years of age, and the mortality rate was 13.8% (4/29).

### Clinical feature and Laboratory results

Eschars were found in 28 (96.6%) patients. With regard to other symptoms, all patients had fever (100%), while 10 with headache, 10 with chills. Lymphadenopathy, rash, vomiting and conjunctival congestion were seen in 6, 5, 2 and 1 instances respectively. All 29 cases were confirmed by Weil–Felix test, of which the genotype Karp type was identified by NPCR in the 4 fatal cases (Table 1).

**Table 1:** **The demographic and clinical features of the 4 death cases**

**Cases**	**Age**	**Gender**	**Onset date**	**History of disease**	**Clinical features**	**Complication**	**Genotype**	**Death date**
1	71	Female	May 16th	Hypertension; hemorrhoids	Fever; headache; rash	severe pneumonia	Karp	May 28th
2	73	Female	May 14th	Hypertension; CHD; pneumonia	Fever; diarrhea; bloody stool	multiple organ function lesion	Karp	May 24th
3	77	Female	May 12th	Hypertension; hemorrhoids	Fever; cough; rash; vomiting	pneumonia	Karp	May 22th
4	57	Male	May 15th	10 years of chest distress	Fever; rash; chilled; convulsion	multiple organ function lesion	Karp	May 22th

### Case–control study

#### Demographic characteristic

Table 2 shows the demographic profiles for the 29 cases and 116 controls. The age group 40–79 years had the predominant number of cases 23/29. Two-thirds of cases had been educated up to junior middle school (Grade 7–9, about 12-15years old). There was only one farmer among the cases. For occupation, 3.4% (1/29) of the cases and 2.6% (3/116) of the controls were farmers. There was no significant difference in gender, age, education and occupation between cases and controls (p > 0.05).

**Table 2:** **Demographic characteristics of the cases and controls in this outbreak**

**Characteristic**	**Case**		**Control**		**P**
	**NO.**	**%**	**NO.**	**%**	
**Gender**					-
Male	14	46.9	56	46.9	
Female	15	53.1	60	53.1	
**Age, year**					-
20-39	4	13.8	12	10.3	
40-59	13	44.8	50	43.1	
60-79	10	34.5	47	40.5	
≥80	2	6.9	7	6	
**Education**					0.371
Junior middle school	20	69	74	63.8	
High school	3	10.3	25	21.6	
Junior college	3	10.3	12	10.3	
Bachelor or above	3	10.3	5	4.3	
**Occupation**					0.800
Farming related	1	3.4	3	2.6	
Non-farming	28	96.6	113	97.4	

### History of outdoor activities and risk behavior

Table 3 shows the crude ORs and adjusted ORs for outdoor activities history and Xiaogang Park activity history. People who took their morning exercise in the park or field within the study period had significantly higher risk (adjusted OR = 3.0, 95% CI: 1.1 - 8.2), People who had activities in Xiaogang Park had a higher risk than those who did not (adjusted OR = 9.1, 95% CI: 3.3 - 25.5). Activities in the Greenery pottery workshop had significantly higher risk of disease (adjusted OR = 4.6, 95% CI: 1.7- 12.5). There was no significant difference between cases and controls with regard to activity in the Picnic place or Shi Ma Gang.

**Table 3:** Activity history, risk factors and protect factor of the scrub typhus in this outbreak*

**Exposure within one month**	**Case (n = 29) No.(%)**	**Control† (n = 116) No.(%)**	**Crude OR(95% CI)**	**Adjusted OR(95% CI)‡**
**Outdoor activity history**				
**Travelling**	24(82.8)	102(87.9)	0.7(0.2,2.0)	0.5(0.2,1.9)
Morning exercise in park or field	22(75.9)	64(55.2)	2.6(1.0,6.4)	3.0 (1.103,8.2)
Activity history in Xiaogang Park	21(72.4)	28(24.1)	8.3(3.8,20.7)	9.1(3.267,25.5)
Activity history in other parks	9(31.0)	28(24.1)	1.4(0.6,3.5)	1.5(0.587,3.8)
**Activity places in Xiaogang Park**				
Picnic place	9(31.0)	34(29.3)	1.1(0.4,2.6)	1.2(0.5,3.1)
Shi Ma Gang	5(17.2)	27(23.3)	0.7(0.2,2.0)	0.7(0.2,2.3)
Greenery pottery work ship	16(55.2)	33(28.4)	3.1(1.3,7.1)	4.6(1.7,12.5)
**Risk behaviors**				
Sitting on lawn	5(18.5)	3(2.8)	8.0(1.8,35.8)	8.0(1.4,44.5)
Fishing	6(20.7)	15(12.9)	1.8(0.6,5.0)	2.2(0.6,7.9)
Close contact with cats	3(10.3)	5(4.3)	2.6(0.6,11.4)	2.4(0.4,13.6)
Close contact with rats	9(31.0)	15(12.9)	3.0(1.2,7.9)	3.3(1.2,9.6)
Sitting near rat holes	7(24.1)	9(7.8)	3.8(1.3,11.2)	6.8(1.2,38.1)
Keep dogs	4(13.8)	14(12.1)	0.9(0.3,2.8)	0.9(0.3,3.0)
Keep cats	3(10.3)	6(5.2)	0.5(0.1,2.0)	0.6(0.1,2.3)
**Protect behaviors**				
Wearing long-sleeved clothing	8(27.6)	62(53.4)	0.3(0.1,0.8)	0.3(0.1,0.7)
Using insect repellent	5(17.9)	22(20.2)	1.2(0.4,3.4)	1.2(0.4,4.0)

*OR = odds ratio; CI = confidence interval.

†Matched by age, sex.

‡Adjusted for age and gender.

For risk behaviors in the adjusted model, sitting on the lawn (adjusted OR = 8.0, 95% CI:1.4 - 44.5), close contact with rats (adjusted OR = 3.3, 95% CI: 1.2 - 9.6) and sitting near the rat holes (OR = 6.8, 95% CI: 1.2 - 38.1) significantly increased the risk for scrub typhus (Table 3). For protective behaviors, wearing long-sleeved clothing while being outdoors was negatively associated with the risk of disease (adjusted OR = 0.3, 95% CI: 0.1 - 0.7), insect repellent use did not show a significant association.

### Response to the epidemic

After 25 May, 2012, the Xiaogang Park was renovated to bring more sunlight and reduce humidity, renovation included lawn mowing and filling of potholes. Pyridaben was used in areas that could not be weeded; bromadiolone was used in the Greenery pottery workshop, picnic place and Shi Ma Gang to control rats. Since the control measures, no new scrub typhus case has been reported in association with the park.

## Discussion

This was an atypical outbreak of scrub typhus associated with a park in Guangzhou. The outbreak, affected 29 people and caused 4 deaths, it was considered unusual as relatively few outbreaks had been previously reported in parks of metropolitan cities. The mortality rate was higher than that reported in South Korea [10], but was similar to that of Thailand [6]. Among the 29 cases, all had fever while eschar was found in 96.5% cases, which was more common than those reported from studies in Japan and Korea [9,10].

The relationship between eschar and mortality was not concordant with a previous report that patients without eschar had > six times risk of severe infection [9]. Though most of our patients had eschars, case fatalities were not negligible (13%).

Karp genotype may have played a critical role in the fatal cases due to its higher virulence compared with other genotypes [11,21–23]. Importantly, all 4 fatal cases had severe chronic conditions, putting them at increased the risk of fatality [24]. The median age of cases was 58, much higher than those reported from other areas [8,9,25], it is to be anticipated that older, particularly elderly, scrub typhus patients will be more likely to develop complications, which may be one of the reasons contributing to the high mortality observed in this outbreak. In addition, since this outbreak occurred before the seasonal epidemic peak of Guangzhou, the time of diagnosis may have been delayed by insufficient awareness among clinicians.

The national surveillance in China showed that the proportion of scrub typhus cases among farmers increased from 58.51% in 2006 to 69.33% in 2012, with farmer to non-farmer ratio of 1:1 in 2006 and 2:1 in 2012 [8]. Research has showed that farmers are the highest risk population for scrub typhus due to long term exposure in fields or orchards. However, barring one, none of our patients had been engaged in agricultural activities. Furthermore, there has recently been an increasing trend of non-farmer cases in Guangzhou; from 172 cases in 2006, to 689 cases in 2012. Further, the percentage of non-farmers among total number of cases was also much higher than the national average, ranging from 44.9% to 51.0% (data unpublished). This suggested that Guangzhou city is becoming a new epidemic focus of scrub typhus, and this infection among non-faming citizenry is gradually increasing. The traditional prevention and control programs often pay great attention to farmers without targeting other occupational groups. This outbreak is a reminder to us that the general public should not be ignored in prevention and control campaign of scrub typhus.

Scrub typhus in parks had been reported in other counties [26–28], but to the best of our knowledge, this is the first report in China. With the rapid development of our economy and urbanization, more people are breaking from their traditional farming or forestry activities. It seems to lower the risk of infection from vector-born infectious disease generally, however, the habitat conditions of scrub typhus includes not only farmlands, but also well-greened places such as grasslands in park. Moreover, park managers priorities may focus on protecting the grassland from damage rather than eliminate potential epidemic area, as a result, unfed chiggers are usually found on dead leaves or low lying vegetation awaiting a suitable host [29,30]. In recent years, rats in the park were found by vector surveillance, the capture rate of rats is as high as 10.4%. The risk of contracting infection from chiggers has increased through ecotourism [31,32] and increasing leisure time in city park. When considering activities and infection risks in parks, we conclude that parks can become epidemic foci for scrub typhus infections.

Our study showed that activity history in the Greenery pottery workshop of Xiaogang Park was a risk factor for scrub typhus. Sitting on the lawn, close contacts with rats and sitting near the rat holes were risk factors and wearing long-sleeved clothing when outside was a protective factor. Use of insect repellent did not show significant protective effects. This suggests that workers exposed during outdoor activities are vulnerable to scrub typhus. Consequently, effective preventive measures should be taken and promoted.

All the cases in this outbreak were investigated at the early stage, thus recall bias was minimized in this case–control study. The disappearance of emerging cases after control measures was a further confirmation of the park as the epidemic focus. Nevertheless, our study is not without limitations. Firstly, our sample size is relatively small which weakens interpretation of the results of the case–control study. Secondly, the 29 non-fatal cases had recovered when we conducted the survey and they refused blood collection, so PCR confirmation was only conducted for the 4 fatal cases. Finally, we were unable to formally demonstrate that infective vectors were present in the park as *Rickettsia ortientalis* was not identified in chiggers which we collected at the last stage of the epidemic.

## Conclusions

In China, outbreaks of scrub typhus are typically reported in rural areas, and farmers are primarily infected. We confirmed an atypical outbreak of Scrub Typhus in a park of Guangzhou, which might develop into an important epidemic site in the city. The risk of infection should not be neglected and we recommend more attention from public health authorities.
